# Ecological Risk and Restoration Measures Relating to Heavy Metal Pollution in Industrial and Mining Wastelands

**DOI:** 10.3390/ijerph16203985

**Published:** 2019-10-18

**Authors:** Huangxin Cheng, Lei Huang, Pengtu Ma, Yi Shi

**Affiliations:** 1 School of Arts and Communication, China University of Geosciences, Wuhan 430000, China or; 2 School of Resources and Environmental Engineering, Hubei University of Technology, Wuhan 430000, China; 3 School of Environmental Studies, China University of Geosciences, Wuhan 430000, China; ptu0912@163.com; 4 Wuhan Zondygreen Environmental Protection Technology Co., Ltd., Wuhan 430000, China; yshi@zdqy.top

**Keywords:** heavy metal, soil, ecological risk, industrial and mining wastelands

## Abstract

In this study, we applied an integrated approach to an ecological risk evaluation of heavy metal pollution in industrial and mining wastelands in Yangxin County, China. A total of 72 sampling sites were designated in the study area. The results show that the potential ecological risk levels of Hg and Cd are higher, and the coefficient of variation of mercury levels is large. Cr, Cu, Zn, Pb, Ni, and As are all at low potential ecological risk. The land types with relatively high ecological risks are alum and coal mines. In the soil of alum mines, the risk due to mercury is higher, while in coal mine soil, the risk due to cadmium is relatively higher.

## 1. Introduction

Heavy metals are of great concern because of their toxicity and persistence in the environment [1]. Heavy metals in soil, such as Hg, Pb, As, Cd, and Cr, can directly poison plants growing nearby as well as the ground and surface water systems, which may also harm human health by enriching agricultural products and water. Consequently, heavy metals are classified among the most dangerous groups of anthropogenic environmental pollutants [2]. Mining and industrial processing are among the main sources of heavy metal contamination in the environment [3]. The ecological risk of heavy metal pollution in soil exists all over the world. Cd, Fe, Pb, and Zn have been found in different plant species in a former mining area in La Union, Spain [4]. Mine tailing and lake sediment from a former mining area in Canakkale, Turkey, were analyzed; the analysis showed that 40%–60% of metals for both mine tailing and sediment were in the residual fraction, and easy mobilization is not expected under the environmental conditions [5]. Devanesan and Gandhi found that coastal sediments from Poombuhar to Karaikal of Tamilnadu are polluted by Ti, V, Cr, Mn, Ca, and Pb due to anthropogenic activities [6]. Sediment samples from the Chodarchay and Gilankesheh rivers, which pass through a mining area in NW Iran, were found to be contaminated by Cd, As, and Pb [7]. According to the national survey bulletin on soil pollution in China, among the 775 soil sites in 81 industrial wastelands surveyed, 34.9% of them exceeded the standard. The main pollutants were Zn, Hg, Pb, Cr, As, and polycyclic aromatic hydrocarbons (PAHs), mainly involving chemical, mining, and metallurgical industries [8]. At present, it is difficult to accurately calculate the total area of industrial and mining wastelands in China. According to some scholars’ estimations, this figure may reach 2 million hectares, of which only 15% has been or is being reclaimed. [9]. Moreover, the main direction in the ecological restoration and reclamation of industrial and mining wastelands in China is toward agricultural land, so investigation of the heavy metal pollution in the soil and ecological risk assessment are very important. It can be predicted that soil pollution assessment and ecological restoration in industrial and mining areas will become increasingly important in China. In this study, we focused on the pollution of heavy metals in soils in various types of industrial and mining wastelands at a regional scale. Typical heavy metals, including As, Cd, Cr, Cu, Hg, Ni, Pb, and Zn, in the soils of industrial and mining wastelands with an area of about 2700 km^2^ in southeastern Hubei Province were sampled and analyzed. On this basis, potential risks in this area were evaluated with the aim to provide a customized scientific basis for the selection of measures to restore soil ecology.

## 2. Materials and Methods

### 2.1. Study Area

The study area was located in the southeastern part of Hubei Province, China. Its geographical coordinates are 29°30′ N~30°9′ N and 114°43′ E~115°30′ E, covering an area of about 2700 square km within Yangxin County. Yangxin County is located in the polymetallic metallogenic belt in the middle reaches of the Yangtze River, with abundant reserves of gold, silver, lead, copper, and zinc as well as coal, limestone, marble, and bentonite. There are 35 proven minerals, comprising 19 metallic minerals and 16 nonmetallic minerals, and 112 mineral deposits. Based on this characteristic, the mineral mining and smelting industry developed very early in this area. Therefore, the problem of heavy metals in the soil of industrial and mining wastelands in this region is representative in the middle reaches of the Yangtze River.

**Figure 1:**
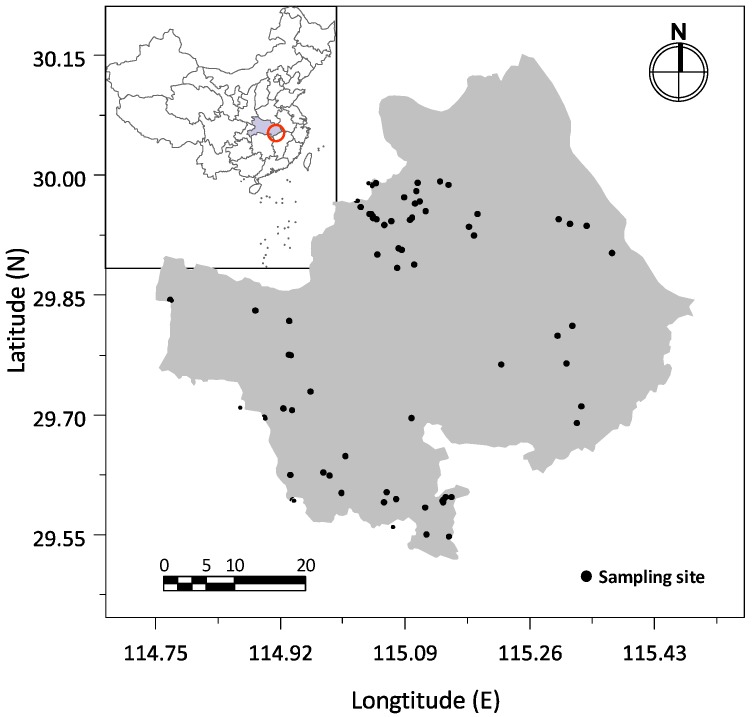
Distribution of soil sampling sites.

In this study, 72 surface soil samples were collected from industrial and mining wastelands in the region. The distribution of sampling points is shown in Figure 1. The land use of before abandonment of these sampling sites was 32 sand and stone mines, 10 brick and tile factories, 9 metal mines, 7 coal mines, 5 bauxite mines, and 9 other industrial and mining wastelands, mainly for building material processing plants.

### 2.2. Soil Sampling and Chemical Analysis

We selected a sampling area of 10 × 10 m in each of the 72 reclaimed industrial and mining wastelands and took five surface soil samples (0–20 cm) with a center point and four diagonal vertices in each sampling area by the plum blossom point method. Threaded drills were used in the sampling. The samples were manually cleared of impurities such as leaves, plant roots, and gravel and uniformly mixed to form a 500 g soil sample representing the sampling area. A total of 72 soil samples were collected. The surface debris was discarded, and the samples were then packaged in polyethylene bags and transferred to the laboratory for analytical testing. The samples were air-dried for 24 h at 60 °C under a fume hood in the laboratory and crushed through a 2 mm nylon mesh. A 5 g sample was taken by multipoint sampling from each of these air-dried samples and was further ground with an agate mortar and passed through a 0.074 mm nylon sieve for use. The soil samples were digested using a HF–HClO_4_–HNO_3_ mixture of 100 mL on an electric heating plate, then diluted to 50 mL using deionized water (18.2 MΩ·cm, resistivity at 25 °C). The pH of the soil samples was determined by the glass electrode method with a water–soil ratio of 2.5:1. The contents of Cd, Cu, Cr, Ni, Pb, and Zn were determined by inductively coupled plasma mass spectrometry (ICP-MS, NexION 350, Perkin Elmer Company, Waltham, MA, USA). The contents of As and Hg were determined using aqua regia digestion and atomic fluorescence spectrometry. The instrument used was a dual-channel atomic fluorescence spectrometer (AFS-830, Jitian Instrument Co., Ltd., Beijing, China). The acid used in the process of analysis and testing was of guaranteed reagent grade (GR), while the other reagents were of analytical reagent grade (AR). Analytical reagent blanks were applied in the sample preparation and analytical processes. All measurements were performed in triplicate, and standard deviations were within ±5% of the mean. The standard reference material was used to control the measurement errors, with recoveries of 100% ± 10% for 8 heavy metals. All metal quantifications were determined according to the Chinese environmental quality standard for soils (GB 15618-1995) and soil quality guidance on sampling techniques (GB/T 36197-2018).

### 2.3. Indexing Approach

#### 2.3.1. Comprehensive Evaluation of Soil Heavy Metal Pollution

The Nemero comprehensive pollution index was applied in this evaluation. The soil heavy metal pollution status of the study area was quantified using the comprehensive evaluation index factor (*P_c_*) approach as follows:

(1) $$P_{c}=\sqrt{\frac{P_{i_{avg}}^{2}+P_{i_{max}}^{2}}{2}},$$

(2) $$P_{i}=\frac{C_{i}}{C_{b}},$$

where *P_c_* is the comprehensive evaluation index factor of soil pollution; *P_i_* is the contamination factor of single heavy metal *i*; *C_i_* is the measured concentration of metal *i*; *C_b_* is the reference value, here the geometric mean of soil metal backgrounds of Hubei province was used [10], as shown in Table 1; and *P_iavg_* and *P_imax_* are the average and maximum values of *P_i_*, respectively.

The comprehensive evaluation index factor (*P_c_*) grading table (Table 2) can be used to judge the pollution degree of heavy metals in soil.

#### 2.3.2. Potential Ecological Risk Evaluation

Common methods suitable for potential ecological risk assessment of soil heavy metal pollution include the geo-accumulation index method, which was proposed by Muller in 1969, and the potential ecological risk index method, which was proposed by Hakanson in 1980. In this study, we attempted to use these two methods to evaluate the ecological risk of soil heavy metals in the study area, and we summarized the results of the two methods to find the general law of the problem.

##### Geo-Accumulation Index

The geo-accumulation index was proposed by the German scientist Muller in 1969 and developed in Europe to study the quantitative indicators of heavy metal pollution in sediments and other materials. When Muller used this method to analyze the degree of contamination of heavy metals, the average value of global shale was chosen as the geochemical background value of the element. The expression of the geo-accumulation index is

(3) $$I_{geo}=\log_{2}\frac{C_{i}}{KC_{b}},$$

where *I_geo_* is the geo-accumulation index of metal *i*; *C_i_* is the measured concentration of *i*; *C_b_* is the soil background value of this metal; and *K* is the variation coefficient of regional differences.

Classification of the geo-accumulation index is determined as shown in Table 3.

##### Potential Ecological Risk Index

The potential ecological risk index is a set of methods for evaluating the potential ecological risk of heavy metals from a sedimentological point of view and based on the toxicity of heavy metals. The expression of the potential ecological risk index is

(4) $$E_{r_{i}}=T_{r_{i}}\frac{C_{i}}{C_{b}},$$

(5) $$RI=\sum_{i=1}^{n}E_{r_{i}},$$

where *E_ri_* is the potential ecological risk index of metal *i*; *T_ri_* is the toxic response factor of metal *i*, demonstrating the metal’s toxic and ecological sensitivity levels (in this study, *T_ri_* takes the value proposed by Hakanson, Table 4); *C_i_* is the measured concentration of metal *i*; *C_b_* is the soil background value of this metal; and *RI* is the integrated potential ecological risk index, calculated as a sum of the *E_ri_* for all examined heavy metals.

The relationships among *RI*, *E_ri_*, and the ecological risk level are shown in Table 5.

## 3. Results and Discussion

### 3.1. Characteristics of Heavy Metal in Soil

The statistics of soil metal concentrations at the sampling sites are shown in Table 6.

According to our results at the 72 sampling points, the concentration of one or more of the metals exceeded the corresponding reference concentration for most sampling points. After calculations according to land types, the ones which exceeded were as follows.

As shown in Table 7, the land use that showed the highest exceedance was “coal mining”, with an average of 75% of the sampled data exceeding the standard, followed by “alum ore mining and processing”, with an average of 63% of the sampled data exceeding the standard. Nearly 50% of sampling data of the other land use also exceeded the standard. Therefore, it can be observed that the accumulation of heavy metal elements in the soil in the study area was widespread, which brings great challenges to soil restoration work in the future.

### 3.2. Comprehensive Evaluation of Soil Heavy Metal Pollution

**Figure 2:**
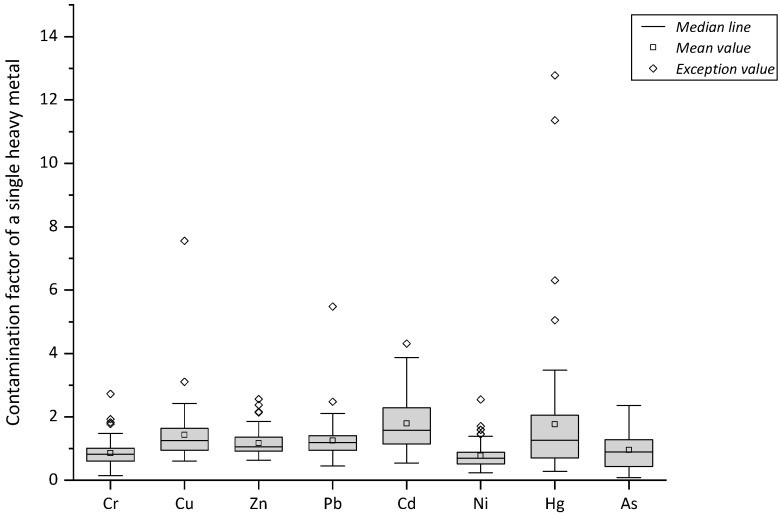
Distribution of the contamination factors of soil heavy metals.

Comprehensive assessment of the soil heavy metal pollution was performed using the comprehensive evaluation index factor of soil pollution. The results are shown in Figure 2 and Table 8.

Relevant studies in the same region of China showed that the pollution index values of Cu, Zn, As, Pb, and Cr in the sediments of Taojiang River in Jiangxi were 3.2, 2.39, 2.17, 1.49, and 0.89, respectively [11]. The pollution index values of heavy metals in the soil in coal mining areas of Bin County, Shaanxi, were Cd, 7.07; Pb, 4.04; Cr, 3.47; Zn, 3.29; and Cu, 0.98 [12]. According to the comprehensive evaluation index factor of soil pollution, the heavy metals examined were present at different degrees of pollution in the study area. The pollution levels of Hg, Cu, Pb, and Cd reached the classification of “severe”. The contamination factor for Hg was extremely high, with a maximum of 12.78, making it the most important heavy metal pollutant within the study area. Cr pollution reached a high level, while those of Zn, Ni, and As reached moderate levels. According to the levels of the comprehensive evaluation index factors, if we ranked them from high to low, the severity of soil heavy metal pollution in the study area was in the order Hg > Cu > Pb > Cd > Cr > Zn > Ni > As.

### 3.3. Potential Ecological Risk Evaluation of Soil Heavy Metal Pollution

#### 3.3.1. Geo-Accumulation Index Evaluation

**Figure 3:**
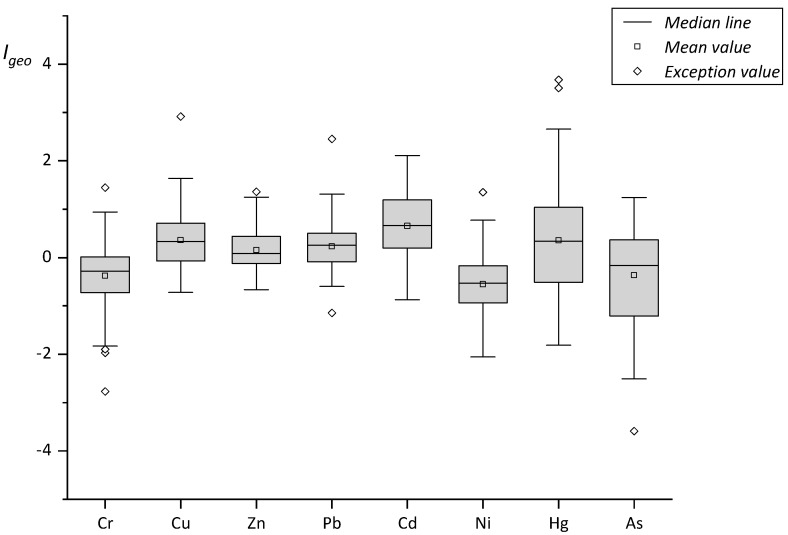
Distribution of the geo-accumulation index (*I_geo_*).

**Figure 4:**
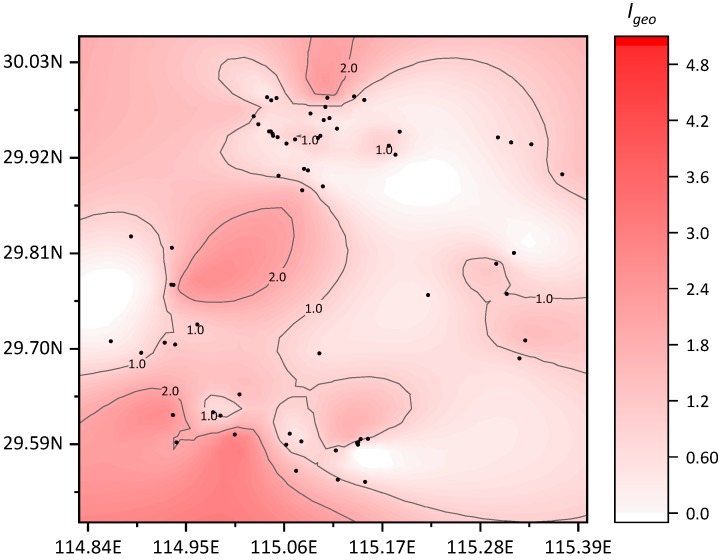
Distribution map of the geo-accumulation index (*I_geo_*).

The geo-accumulation index (*I_geo_*) of each metal element was calculated according to the geo-accumulation evaluation proposed by Muller. The values of *C_b_* are shown in Table 1, and the value of *K* was 1.5. The range and distribution of the geo-accumulation index values of the eight heavy metals in the soil samples from the study area are shown in Figure 3. The calculated geo-accumulation indices with average and standard deviation is show in Table 9. A contour map of the geo-accumulation index values of each sampling point in the area is shown in Figure 4.

As shown in Figure 3 and Table 9, according to the distribution of the geo-accumulation index values of the eight heavy metals, the mean value was between −0.55 and +0.66. Relative to the various metal elements, the numbers of sampling points at different ecological risk levels are shown in Table 10.

In the study area, the land uses with higher ecological risks were alum and coal mines. Within the soil samples of these areas, most of the ecological risk level of metals moved towards medium pollution. In the ore soil, the risk due to Hg levels was higher, while in coal mine soil, the risk due to Cd was higher. There were 5 soil metal I_geo_ values between 2.0 and 3.0, which included one alum mining site, 2.11 (Cd); one brick and tile site, 2.66 (Hg); two other sites, 2.34(Hg) and 2.46(Hg); and one sandstone site, 2.92 (Cu). Two samples were above 3.0, 3.51 (Hg) and 3.68 (Hg), which were located in two sandstone sites.

Relevant studies in China showed that the pollution risk level of Pb in farmland soils in the typical lead–zinc mining area of Yueyang City, Hunan Province, was close to the level of medium pollution, while the risk grades of As, Cd, Cu, Ni, and Zn were at nonpollution levels [13]. The *I_geo_* values of 15 sampling sites were between 0.29 and 1.37 in Yuqiao, Tianjing city [14]. A study in Yong’an coal mining area showed that the *I_geo_* value of Cr was less than 0, and the *I_geo_* values of Zn, As, Pb, Cu, and Cd were between 0 and 1 [15].

#### 3.3.2. Potential Ecological Risk Index Evaluation

**Figure 5:**
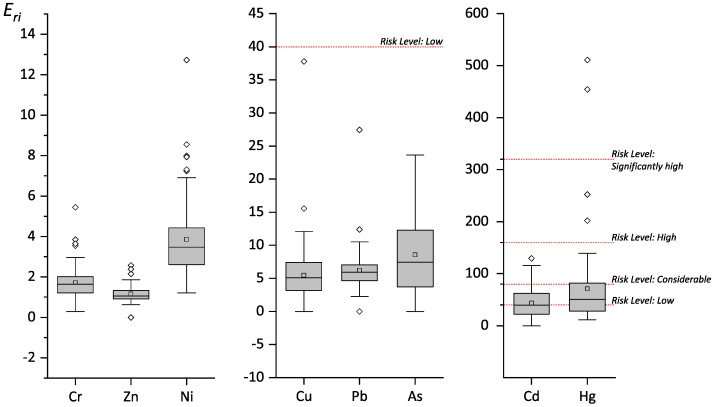
Distribution of the potential ecological risk index (*E_ri_*).

**Figure 6:**
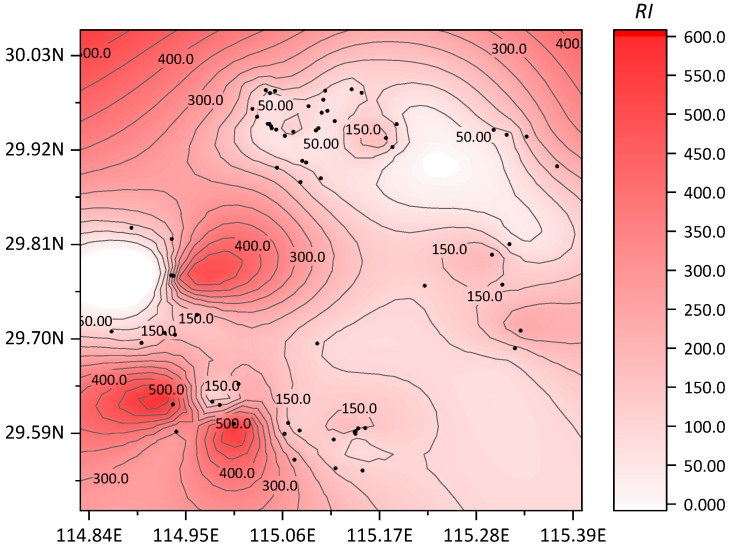
Distribution map of the integrated potential ecological risk index (*RI*).

According to the potential ecological risk index evaluation proposed by Hakanson, the individual potential ecological risk coefficients (*E_ri_*) and the comprehensive potential ecological risk index (*RI*) values were calculated. By combining them with the potential ecological risk grading standard (Table 5), the potential ecological risk assessment results of heavy metals in reclaimed soil were obtained, as shown in Figure 5, Figure 6 and Table 11.

The ranking of the average value of the potential ecological risk index (*E_ri_*) of the eight heavy metals in the reclaimed soil was Hg > Cd > As > Pb > Cu > Ni > Cr > Zn. From the overall situation of the 72 sampling points, Cr, Cu, Zn, Pb, Ni, and As had low potential ecological risks, and there were no data exceeding this level. Risk levels for Cd were considered as low (37 sites), moderate (25 sites), and considerable (10 sites). The coefficient of variation of Hg was the largest among the eight heavy metals, and its spatial variability was the strongest. Therefore, the potential ecological risk level of Hg varied greatly across low risk (27 sites), moderate risk (27 sites), considerable risk (15 sites), high risk (2 sites), and significantly high risk (2 sites). According to the land use, these were mainly distributed in alum and coal mines. A high coefficient of variation of Hg was also found in areas with high industrialization and urbanization in southern China. He Bo found that the coefficient of variation of Hg in the topsoil from their study area reached 69.23%, which was the highest among all metals tested. That soil was considered more likely to be affected by external factors [16].

According to the calculations, the comprehensive potential ecological risk index (*RI*) values of the reclaimed soils ranged from 37.64 to 556.06, with an average value of 140.84. Among them, there were only 47 sampling points at low potential ecological risk, accounting for 65.3% of the total sampling points; 22 sampling points at moderate risk, accounting for 30.5%; 3 sampling points at high risk, accounting for 4.2%; and no sampling points at the “severe” risk level. Relevant studies in other regions of China showed that the *RI* values of reclaimed soils in a Huangshi abandoned quarry ranged from 112.49 to 363.62 [17]. The *RI* values of reclaimed soils in Antaibao Open-pit Coal Mine in Pingshuo ranged from 131.43 to 331.03, with an average of 191.68 [18]. The average *RI* value of reclaimed soils in a Shanghai reclaimed industrial site was 385.79 [19]. The average *RI* of Shunde waterway soil was 73.20 [20]. The average RI of farmland soil around a tungsten mining area in Xianghualing, Hunan Province was 330.4 [21]. The range of the *RI* of farmland soil around a smeltery in Hunan Province was 46.4–1627.5 [22]. The variation range of the *RI* of soil from Hengyang Songjiang Industrial Park was 330.51–17,721.89, with an average of 2374.32 [23]. Compared with other reclaimed soils or other types of soils in other areas, the potential ecological risk of reclaimed soils in this study was generally at a relatively moderate level. However, because of the high ecological toxicity of Cd and Hg, the potential ecological risk of some reclaimed soils was higher, and these areas should be monitored.

#### 3.3.3. A Discussion of the Potential Ecological Risk Evaluation of Soil Heavy Metal. Pollution

Based on the results of the geo-accumulation index evaluation and potential ecological risk index evaluation, both methods showed that the potential ecological risk of soil heavy metals in the study area is generally at a low level, and it is feasible to carry out reclamation. Common engineering techniques for soil ecological restoration include physical improvement, chemical improvement, and biological improvement. For mildly contaminated soils, the use of chelating agents and super-accumulated plants for purification can be considered [24]. In the study area, Hg and Cd are the main potential ecological risk factors in the soil, and it is necessary to pay attention to the fact that the potential ecological risk value of Hg in gravel ore is extremely high. For soils that are moderately or heavily contaminated with heavy metals, a faster method is to replace the soil. Studies have shown that after removing 15 to 30 cm of soil on the surface and filling in the area with clean soil, the concentration of heavy metals in plants can be reduced by more than 50% [25]. However, this method is very expensive when used to repair large areas of contaminated soil. Soil leaching and soil thermal desorption are also feasible methods. Pociecha and Lestan used electrocoagulation to recover heavy metals from a leaching solution of EDTA and contaminated soil and found that this method can remove 53% of Pb, 26% of Zn, and 52% of Cd from contaminated soil [26]. Studies by Kunkel and some others have shown that in situ thermal desorption can remove 99.8% of Hg from contaminated soils at temperatures below the boiling point of the soil [27].

## 4. Conclusions

Both geo-accumulation index evaluation and potential ecological risk index evaluation are useful tools for heavy metal ecological risk evaluation. They can effectively reflect the outstanding risk issues of the study area. As a case study, an integrated approach was applied to an ecological risk evaluation of heavy metal pollution in industrial and mining wastelands of Yangxin County, China. A total of 72 sampling sites and 8 metallic elements, As, Cd, Cr, Cu, Hg, Ni, Pb, and Zn, were included in this potential ecological risk study. Both methods showed that the potential ecological risk of soil heavy metals in the study area cannot be ignored. This may mean that future reclamation of this land will be costlier. Among the elements examined, the potential ecological risk levels of Hg and Cd are higher, and the coefficient of variation of Hg content is large. Cr, Cu, Zn, Pb, Ni, and As are all at low risk. The land uses with relatively high ecological risks are alum and coal mines. In the soil of alum mines, the risk of Hg is higher, while in coal mine soil, the risk of Cd is relatively higher. Therefore, Hg and Cd pollution is generally significant in this study area, which is worthy of future vigilance.

## Figures and Tables

**Table 1:** Background soil heavy metals in the study area.

Element	pH	Cr	Cu	Zn	Pb	Cd	Ni	Hg	As
*C_b_* (mg/kg)	6.5	79.0	28.2	77.5	25.7	0.1137	38.6	0.0634	10.5

**Table 2:** Grading of soil heavy metal pollution.

*P_c_*	Pollution Level
*P_c_* ≤ 0.7	Clean
0.7 < *P_c_* ≤ 1.0	Low
1.0 < *P_c_* ≤ 2.0	Moderate
2.0 < *P_c_* ≤ 3.0	High
*P_c_* > 3.0	Severe

**Table 3:** Classification of the geo-accumulation index.

Classification	*I_geo_*	Risk Level
0	*I_geo_* < 0	Nonpollution
1	0 ≤ *I_geo_* < 1	Nonpollution to Medium pollution
2	1 ≤ *I_geo_* < 2	Medium pollution
3	2 ≤ *I_geo_* < 3	Medium pollution to Serious pollution
4	3 ≤ *I_geo_* < 4	Serious pollution
5	4 ≤ *I_geo_* < 5	Serious pollution to Extremely serious pollution
6	*I_geo_* ≥ 5	Extremely serious pollution

**Table 4:** Toxic response factors of heavy metals.

Element	Cr	Cu	Zn	Pb	Cd	Ni	Hg	As
*T_r_*	2	5	1	5	30	5	40	10

**Table 5:** Classification of the potential ecological risk index and integrated potential ecological risk index.

Scope of Potential Ecological Risk (*E_ri_*)	Risk Level	Scope of Integrated Potential Ecological Risk (*RI*)	Risk Level
*E_ri_* < 40	Low	*RI* < 150	Low
40 ≤ *E_ri_* < 80	Moderate	150 ≤ *RI* < 300	Moderate
80 ≤ *E_ri_* < 160	Considerable	300 ≤ *RI* < 600	High
160 ≤ *E_ri_* < 320	High	*RI* ≥ 600	Severe
*E_ri_* ≥ 320	Significantly high		

**Table 6:** Concentration statistics of heavy metals in soil.

Elements	Background *C_b_* (mg/kg)	Concentration *C_i_* (mg/kg)		Coefficient of Variation
		Range	Average	
Cr	79	11.60–215.00	67.96 ± 32.86	48.02%
Cu	28.2	17.10–213.00	40.38 ± 27.37	67.17%
Zn	77.5	48.90–199.00	90.71 ± 30.61	33.51%
Pb	25.7	11.60–141.00	32.37 ± 16.14	49.51%
Cd	0.1137	0.062–0.490	0.204 ± 0.105	51.26%
Ni	38.6	9.28–98.30	29.79 ± 15.92	53.08%
Hg	0.0634	0.018–0.810	0.112 ± 0.129	114.67%
As	10.5	0.870–24.800	10.085 ± 6.162	60.62%

**Table 7:** The number of sampling points whose concentration exceeds the reference and its land use type.

Types of Land Use Before Disposal	Number of Sampling Points	Number of Sampling Points Whose Concentration Exceeds the Reference Value							
		Cr	Cu	Zn	Pb	Cd	Ni	Hg	As
Sandstone Ore Mining and Processing	32	7	17	18	18	19	6	18	9
Brick and tile factory	10	1	5	3	8	8	0	10	3
Metal ore mining and processing	9	1	4	9	4	6	0	1	1
Coal mining	7	4	5	4	7	5	3	7	7
Alum ore Mining and Processing	5	3	4	3	4	2	2	5	2
Other Industrial Wasteland	9	2	3	5	7	7	2	4	5

**Table 8:** Results of the comprehensive evaluation of soil heavy metal pollution.

Elements	Contamination Factor *P_i_*		Comprehensive Evaluation Index *P_c_*	
	Range	Average	Value	Pollution Level
Cr	0.15–2.72	0.86 ± 0.42	2.02	High
Cu	0.61–7.55	1.43 ± 0.97	5.44	Severe
Zn	0.63–2.57	1.17 ± 0.4	2.00	Moderate
Pb	0.45–5.49	1.26 ± 0.63	3.98	Severe
Cd	0.55–4.31	1.79 ± 0.93	3.30	Severe
Ni	0.24–2.55	0.77 ± 0.41	1.88	Moderate
Hg	0.28–12.78	1.77 ± 2.04	9.12	Severe
As	0.08–2.36	0.96 ± 0.59	1.80	Moderate

**Table 9:** Values of the geo-accumulation index (*I_geo_*).

Elements	Geo-Accumulation Index *I_geo_*	
	Range	Average
Cr	−2.77–1.44	−0.38 ± 0.71
Cu	−0.72–2.92	0.36 ± 0.61
Zn	−0.66–1.36	0.16 ± 0.44
Pb	−1.15–2.46	0.23 ± 0.51
Cd	−0.87–2.11	0.66 ± 0.74
Ni	−2.06–1.35	−0.55 ± 0.73
Hg	−1.82–3.68	0.35 ± 1.06
As	−3.59–1.24	−0.37 ± 1.02

**Table 10:** Distribution of sampling points with different risk levels under ecological risk assessment by the geo-accumulation index.

Elements	Number of Sampling Sites				
	Nonpollution	Nonpollution to Medium Pollution	Medium Pollution	Medium Pollution to Serious Pollution	Serious Pollution
Cr	54	17	1	0	0
Cu	34	32	5	1	0
Zn	30	38	4	0	0
Pb	25	43	4	1	0
Cd	25	28	18	1	0
Ni	59	12	1	0	0
Hg	27	26	15	2	2
As	45	22	5	0	0

**Table 11:** Values of the potential ecological risk index (*E_ri_*).

Elements	Potential Ecological Risk Index *E_ri_*	
	Range	Average
Cr	0.29–5.44	1.72 ± 0.83
Cu	0.00–37.77	5.47 ± 5.22
Zn	0.00–2.57	1.15 ± 0.42
Pb	0.00–27.43	6.21 ± 3.21
Cd	0.00–129.29	43.28 ± 32.83
Ni	1.20–12.73	3.86 ± 2.06
Hg	11.36–511.04	70.61 ± 81.54
As	0.00–23.62	8.54 ± 6.31

## References

[1] Lv J., Liu Y. (2019). An integrated approach to identify quantitative sources and hazardous areas of heavy metals in soils. Sci. Total Environ..

[2] Zhu D., Wei Y., Zhao Y., Wang Q., Han J. (2018). Heavy Metal Pollution and Ecological Risk Assessment of the Agriculture Soil in Xunyang Mining Area, Shaanxi Province, Northwestern China. Bull. Environ. Contam. Toxicol..

[3] Boamponsem L.K., Adam J.I., Dampare S.B., Nyarko B.J.B., Essumang D.K. (2010). Assessment of atmospheric heavy metal deposition in the Tarkwa gold mining area of Ghana using epiphytic lichens. Nucl. Instrum. Methods Phys. Res. Sect. B.

[4] Lambrechts T., Couder E., Bernal M.P., Faz Á., Iserentant A., Lutts S. (2010). Assessment of Heavy Metal Bioavailability in Contaminated Soils from a Former Mining Area (La Union, Spain) Using a Rhizospheric Test. Water Air Soil Pollut..

[5] Karaca O., Cameselle C., Reddy K.R., Yesiller N., Zekkos D. (2016). Characterization of Heavy Metals in Mine Tailings and Lake Sediments: Implications on Remediation. Geo-Chicago 2016: Sustainable Waste Management and Remediation.

[6] Devanesan E., Suresh Gandhi M., Selvapandiyan M., Senthilkumar G., Ravisankar R. (2017). Heavy metal and potential ecological risk assessment in sedimentscollected from Poombuhar to Karaikal Coast of Tamilnadu using Energy dispersive X-ray fluorescence (EDXRF) technique. Beni Suef Univ. J. Basic Appl. Sci..

[7] Yasami N., Ghaderi M., Taghilou B. (2019). Heavy metal assessment in stream sediments from the rivers passing through the mining area. Int. J. Environ. Sci. Technol..

[8] Ministry of Education of the People’s Republic of China (2014). China National Survey Bulletin on Soil Pollution.

[9] Zhu D., Yang Q. (2018). Study on current situation and ecological risk of heavy metal pollution in mining areas of China. Miner. Explor..

[10] Center C.N.E.M. (1990). China’s Soil Element Background Values.

[11] Chen M., Hu L., Tao M., Li F., Shi Y. (2019). Heavy metal pollution characteristics and risk assessment in sediment of Taojiang river. Acta Sci. Circumstantiae.

[12] Chen Y. (2017). Pollution and ecological risk assessment of heavy metals in soil of western Shaanxi province. Environ. Dev..

[13] Guo Z., Tu W., Peng C., Huang B., Xiao X., Xue Q. (2017). Distribution characteristics and potential ecological risk assessment of heavy metals in paddy soil along both sides of river from typical lead/zinc mine area. J. Agro-Environ. Sci..

[14] Wang Q., Wang Z., Hou Y., Wang Z. (2018). Application of improved geo-accumulative index method in the ecological assessment of heavy metals. J. Tianjin Norm. Univ..

[15] Lu J., Huang Y., Wang C., Cheng X., Liu R. (2018). Evaluation of Heavy Metals Pollution to Soil and Air Dust Near Ground of Yong’an Coal Mining Area in Jilin. Ind. Saf. Environ. Prot..

[16] He B., Zhao H., Wang T., Meng J., Xiao R. (2019). Spatial Distribution and Risk Assessment of Heavy Metals in Soils from a Typical Urbanized Area. Environ. Sci..

[17] Yin X., Tang L., Zeng Q., Bai L., Zhao Y., Yang X. (2018). Distribution and Pollution Assessment of Heavy Metals in Soils After Artificial Vegetation Restoration in Abandoned Quarries in Huangshi. Hubei For. Sci. Technol..

[18] Deng H., He W., Zhou K. (2015). Heavy metals distribution in reclamation tailings and assessment of ecological risk. Chin. J. Nonferrous Met..

[19] Wu J., Wang M., Zhang H., Huang Y., Xu Z., Li Q., Chen H., Huang S. (2018). Heavy Metal Pollution and Potential Ecological Risk of Soil from Reclaimed Industrial Sites and Surrounding River Sediments. Environ. Sci..

[20] Cai Y., Chen W., Peng C., Wang T., Xiao R. (2016). Spatial Distribution and Potential Ecological Risk Assessment of Heavy Metals in Soils and Sediments in Shunde Waterway, Southern China. Environ. Sci..

[21] Zhang Y., Hu J., Liu J., Chen H., Yang X., Zhao Z., Liu F. (2018). Pollution Characteristics and Ecological Risks of Heavy Metals in the Soil from Xianghualing Tungsten Mining Area of Hunan Province. Safty Environ. Eng..

[22] Lu S., Wang Y., He L. (2015). Heavy Metal Pollution and Ecological Risk Assessment of the Paddy Soils near a Smelting Area in Hunan Province. Environ. Monit. China.

[23] Peng Y., Qiu G., Jiang H., Wang Y. (2013). Study on the Soil Heavy Metal Pollution in Songjiang Industrial Park of Hengyang City. Safty Environ. Eng..

[24] Hou S., Wang X., Liu D. (2019). Study on ecological restoration and landscape construction technology of urban heavy metal contaminated soil. J. Green Sci. Technol..

[25] Wang X., Wang C., Wu X., Wang J., Mu X., Yang X., Hu X., Gao J. (2019). Research Progress in Remediation Technology of Heavy Metal Contaminated Soil. Chem. Bioeng..

[26] Pociecha M., Lestan D. (2010). Using electrocoagulation for metal and chelant separation from washing solution after EDTA leaching of Pb, Zn and Cd contaminated soil. J. Hazard. Mater..

[27] Kunkel A.M., Seibert J.J., Elliott L.J. (2006). Remediation of elemental mercury using in situ thermal desorption (ISTD). Environ. Sci Technol..

